# The relationship between unpredictability in childhood and depression among college students: the mediating roles of coping style and resilience

**DOI:** 10.1186/s40359-024-01812-8

**Published:** 2024-06-06

**Authors:** Chengxiu Ye, Baojuan Ye, Zheng Zhang

**Affiliations:** 1 School of Psychology, Jiangxi Normal University, Nanchang, China; 2 Department of Applied Psychology, Steinhardt School of Culture, Education and Human Development, New York University, New York, NY USA; 3 Department of Psychiatry, National Center for Mental Disorders, National Clinical Research Center for Mental Disorders, The Second Xiangya Hospital of Central South University, Changsha, Hunan China; 4 School of Physical Education, School of Education, Jiangxi Normal University, Nanchang, China

**Keywords:** Unpredictability in childhood, Coping style, Resilience, Depression, Chinese college students

## Abstract

**Background:**

According to previous studies, unpredictability in childhood could significantly increase the risk of depression in adulthood. Only a few studies have explored the relationship between these two variables in China. This paper aims to explore the relationship between unpredictability in childhood and depression and examine the mediating roles of coping styles and resilience.

**Methods:**

We investigated 601 college students, who had an average age of 19.09 (SD = 2.78) years. Participants completed questionnaires regarding unpredictability in childhood, coping style, resilience, and depression. We analyzed survey data using the bias-corrected bootstrap method.

**Results:**

The findings revealed a significant positive association between unpredictability in childhood and depression among college students. Mature coping style, immature coping style, and resilience were found to mediate this relationship independently. Furthermore, the study unveiled a serial mediation process, wherein both mature and immature coping styles, followed by resilience, sequentially mediate the relationship between unpredictability in childhood and depression, underscoring the complex interplay between these variables.

**Conclusions:**

The results indicated that the risk of depression among college students who have experienced unpredictable childhood should be valued. Attention to coping styles and resilience should be paid to decrease depression among college students who have experienced unpredictable childhood.

## Introduction

Depression is a highly prevalent mental disorder worldwide, with an estimated lifetime prevalence of between 8% and 12% [1]. According to the World Health Organization, it is also the leading cause of lifelong disability [2]. While some studies have examined the impact of early family environment on depression [3, 4], only a few have focused on the relationship between unpredictability during childhood and individual mental health in China [5, 6], with no research specifically exploring the link between unpredictability in childhood and depression among Chinese college students. Nevertheless, animal studies have demonstrated a correlation between unpredictability during childhood and the development of emotional and cognitive brain functions, ultimately affecting mental health [7]. Additionally, other studies have shown that unpredictability during childhood is associated with an increased risk of depression in adulthood [8, 9]. Although the association between unpredictability in childhood and depression was shown in previous studies, it is still unclear how these psychological factors, like mature coping style, immature coping style, and resilience mediate the pathway through which the unpredictability in childhood impacts depression. Given the significant impact of depression and the current paucity of research exploring the link between unpredictability in childhood and depression within the Chinese context, it is crucial to investigate the mechanism behind the relationship between unpredictability in childhood and depression.

### Unpredictability in childhood and depression

Based on the theory of evolutionary advantages of depression, depression might be an adaptive skill of individuals to prevent wasting their own energy in some adverse environments, since efforts might bring danger, loss, and waste of energy in these environments [10]. Moreover, the unpredictable early environment, as a kind of adverse environment, may be related to depression. Studies have shown that when an infant is in an unpredictable environment, unpredictable signals emanating from the mother affect his or her brain, eventually adversely affecting the development of the cognitive and emotional system of the individual brain [7]. Some researchers put mice in an environment where their mothers sent unpredictable signals to their young. After these young mice had grown up, they exhibited a decline in memory [11, 12] and a lack of pleasure experience [13]. In terms of research with humans, the unpredictable behavior and emotion of the mother would increase the risk of mental illness of the child [14–16]. Additionally, the unpredictable living environment in the early stage has a significant correlation with depression in adulthood [9]. Therefore, unpredictability in childhood may be positively correlated with depression in college students.

### The mediating role of coping style in the relationship between unpredictability in childhood and depression

Coping style is a certain cognitive and behavioral adaptation effort when individuals face some heavy pressure beyond their psychological compatibility [17]. It plays an important role for individuals when they are confronted with stressful life events, and specific coping styles might be positively or negatively correlated with mental states [18]. Coping styles, fundamental to psychological resilience and response to stress, can be broadly categorized into mature and immature types based on their effectiveness and adaptiveness in addressing stressors [19, 20]. Mature coping strategies, such as problem-solving, seeking social support, and positive reappraisal, are characterized by their proactive and constructive approach to dealing with stress, promoting adaptation and psychological well-being. Conversely, immature coping styles, including avoidance, denial, and emotional venting, are generally considered less adaptive, often exacerbating stress and hindering resolution [19–21]. The classification of coping strategies into mature and immature is rooted in their observed outcomes on psychological health across diverse situational contexts, acknowledging that the efficacy of a coping strategy can indeed be contingent upon the nature of the stressor and the individual’s circumstances [19, 20]. This differentiation is pivotal in understanding the mechanisms through which childhood unpredictabilty influences depression.

According to life history theory, in an unpredictable living environment, disease or violence might taint the value of long-term investments. In such an environment, individuals cannot obtain any returns, so individuals are more inclined to pursue immediate gratification [22], and this tendency to seek instant gratification may lead individuals to adopt immature coping styles. Research has shown that early family environment is correlated with adults’ coping styles [23], and lots of studies have verified the influence of early life environment on individual adaptation function in the future [24]. Therefore, it is believed that unpredictability in childhood was correlated with individuals’ coping styles in this study. Meanwhile, previous studies have shown that coping styles are positively or negatively correlated with depression [25, 26]. Immature coping style is positively correlated with depression, while mature coping style is negatively correlated with depression [27]. College students who adopted mature coping styles could cope with pressure maturely and had less depression [28], while individuals who adopted immature coping styles might have an increased risk of depression [25, 29]. Therefore, mature coping style and immature coping style may mediate the relationship between unpredictability in childhood and depression.

### The mediating role of resilience in the relationship between coping style and depression

Resilience refers to the positive adjustment process of individuals to achieve mental and physical balance in the face of adverse events or traumatic events [30]. According to cognitively oriented theory, individuals can obtain better assistance to face the pressure caused by adverse events by identifying and changing the immature coping styles in their thoughts and behaviors [31]. Studies have shown that mature coping styles (problem-solving, planning, etc.) are ways of coping with adverse events and are related to better outcomes; instead, immature coping styles (denial, venting, etc.) are always related to bad results when individuals are dealing with stressful events [32, 33]. Therefore, different coping styles may be related to different levels of resilience: individuals who adopt mature coping styles can solve the problems they face through effective behaviors, thereby enhancing resilience and vice versa. At the same time, several previous studies have found that resilience was significantly negatively correlated with depression. Individuals with a high level of resilience are less prone to depression [34–36]. Therefore, mature coping styles might make individuals less prone to depression by enhancing their resilience. Instead, immature coping styles may make individuals prone to depression by reducing their resilience. Therefore, resilience may be a mediating variable between mature coping style, immature coping style, and depression.

### The present study

**Fig. 1:**
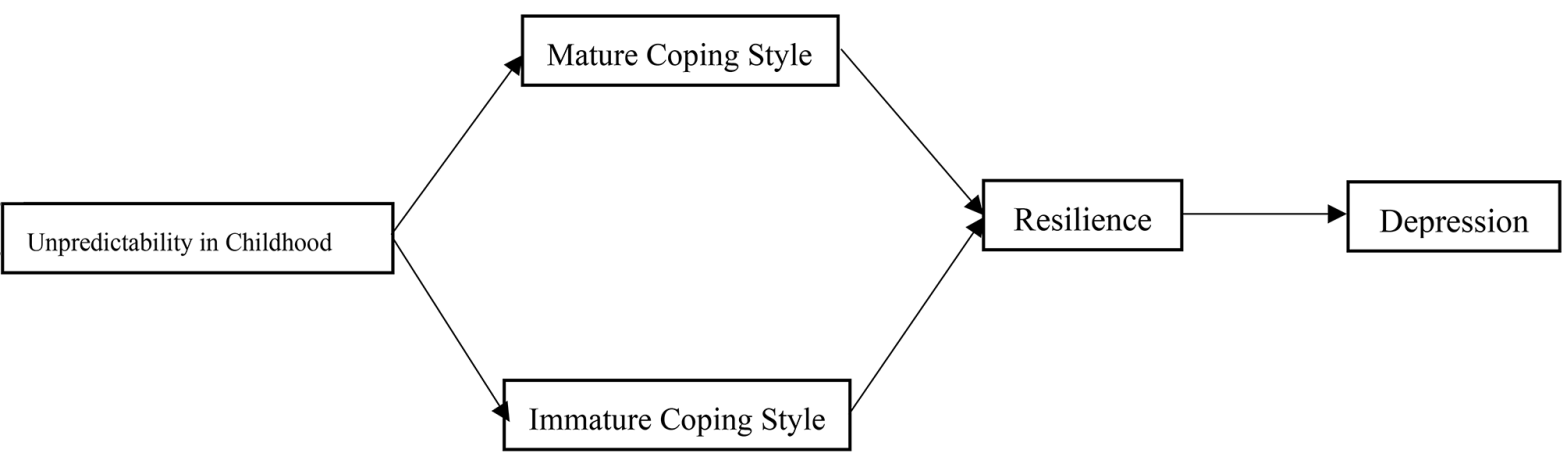
Conceptual framework diagram

This study constructed a serial mediation model (see Fig. 1) to test the mediating effect of mature coping style, immature coping style, and resilience. Based on existing research conclusions and theories, this study proposes hypotheses:

#### Hypothesis 1

Unpredictability in childhood is positively correlated with depression among college students.

#### Hypothesis 2

Mature coping style and immature coping style mediate *the relationship* between unpredictability in childhood and depression.

#### Hypothesis 3

Resilience mediates the relationship between mature coping style and depression. Resilience mediates the relationship between immature coping style and depression.

## Method

### Participants

In this study, a total of 647 students were initially enrolled to participate in the survey. Of these, 601 participants (54.2% of whom were female) met the inclusion criteria, resulting in a completion rate of 92.89%. The data collection was conducted through paper-based questionnaires. Before distributing the surveys, participants underwent an informed consent procedure, ensuring they were fully aware that their involvement was entirely voluntary, uncompensated, and could be terminated at any moment. Among the total sample, 185 (30.7%) were first years, 224 (37.3%) were second years, 129 (21.5%) were third years, and 72 (12.0%) were fourth years. The mean age of the participants was 19.09 (SD = 2.78, range = 17–23).

### Measures

#### Questionnaire of unpredictability in childhood

Unpredictability in Childhood was assessed with the Questionnaire of Unpredictability in Childhood [9]. It went through multiple translation and back-translation procedures conducted by researchers proficient in both English and psychology. In the initial questionnaire development phase, the scale was refined through exploratory factor analysis using SPSS 20.0 with a sample of 400 participants. After the analysis, a final set of 10 items was retained, each of which was rated as either “yes” or “no”. The scale encompasses three distinct subscales: parental life participation, parental emotional stability, and parental supervision and inspection. In a subsequent phase, the QUIC was administered to 603 participants. The Cronbach’s alpha of the scale in this study was 0.73. The Cronbach’s alpha for the subscales were 0.69, 0.63, and 0.70, respectively. This scale has good construct validity in this study (χ2/df = 3.34, TLI = 0.91, CFI = 0.94, RMSEA = 0.06, SRMR = 0.05).

#### Center for epidemiological survey, depression scale

Depression was assessed with the Center for Epidemiological Survey, Depression Scale [37]. This scale has been used among the Chinese participants before [38]. There are 20 items and each item was rated on a 4-point scale (0 = occasionally or none to 3 = most of the time or continuously), with higher total scores indicating greater depression. The Cronbach’s alpha of the scale in this study was 0.86.

### Ego resiliency scale

Resilience was assessed with the Ego Resiliency Scale [39]. This scale has been used among Chinese participants before [40]. It was scored on a 4-point scale (1 = completely inconsistent” to 5 = completely consistent), with higher total scores indicating a higher level of resilience. Studies have shown that the scale has good reliability and validity in China [40–42]. In this study, the Cronbach’s alpha of the scale was 0.83.

### Coping style questionnaire

Mature coping style and immature coping style were assessed using the Coping Style Questionnaire developed by Xiao [43]. This questionnaire includes 62 items, with responses recorded as either “yes” or “no”, and comprises six subscales: problem-solving, self-blame, seeking help, retreat, fantasy, and rationalization. Following the classification in previous research, these subscales are grouped into two overarching categories: a mature coping style, encompassing problem-solving and seeking help, and an immature coping style, including self-blame, fantasy, retreat, and rationalization [44, 45]. This division is based on the demonstrated impact these styles have on psychological well-being.

The selection of the Coping Style Questionnaire was motivated by its proven reliability and validity in the context of Chinese populations [44–46]. In our research, Cronbach’s alpha values were 0.84 for the immature coping style scale and 0.71 for the mature coping style scale, indicating satisfactory internal consistency.

### Procedure

The study was approved by the ethics committee of the first author’s university. In this study, participants over the age of 18 provided informed consent. Group testing was adopted, emphasizing the principles of anonymous filling, data confidentiality, and voluntary filling. There was no compensation for participating in this study, and the participants participated entirely voluntarily.

### Analytical strategy

This study has adopted IBM SPSS 23.0 and Mplus 8.3 statistical software for all data analyses. After the questionnaires were collected, all the data were processed as follows: (1)SPSS 23.0 was used to calculate the descriptive statistics for the study variables, and then their correlation between the study variables was calculated. (2)According to the mediating effect test procedure proposed by previous researchers [46], hierarchical regression analysis was used to analyze the mediating role of coping style and resilience between unpredictability in childhood and depression. (3)Mplus 8.3 was used to repeatedly sample 2000 times with the bootstrap method to calculate the 95% confidence interval, mediating effect, and total effect.

## Results

Following the mediation effect testing procedure [47, 48], this study delves into the mediating roles of mature coping style, immature coping style, and resilience in the relationship between unpredictability in childhood and depression. In our study, we employ sequential regression analysis to test for mediation effects. For a mediation effect to be deemed significant, it must satisfy three conditions: (1) The independent variable (e.g., unpredictability in childhood) must significantly correlate to the dependent variable (e.g., depression); (2) The independent variable (e.g., unpredictability in childhood) must significantly correlate to the mediating variables (e.g., mature coping style, immature coping style, and resilience); and (3) These mediating variables (e.g., mature coping style, immature coping style, and resilience) in turn significantly correlate with the dependent variable (e.g., depression). This can be ascertained through sequential regression or by employing bootstrap methods for a determination of mediation effects. Given that sequential regression allows for a more detailed exploration of each mediation pathway’s significance compared to bootstrap methods, our analysis has incorporated both approaches to ensure a comprehensive examination of mediation [47]. Aside from gender, all variables were standardized. Data processing using Harman’s single-factor test revealed that common method variance did not pose a significant issue in this investigation.

### Correlation analysis between variables

Table 1 provides the Pearson correlations for the study variables. As the results show, unpredictability in childhood and immature coping style was positively correlated with depression (*r* = 0.22, *p* < 0.01 and *r* = 0.50, *p* < 0.01, respectively) while mature coping style and resilience were significantly negatively correlated with depression (*r*=-0.41, *p* < 0.01 and *r*=-0.32, *p* < 0.01, respectively). Unpredictability in childhood was negatively correlated with mature coping style and resilience (*r*=-0.23, *p* < 0.01 and *r*=-0.18, *p* < 0.01, respectively), and positively correlated with immature coping style (*r* = 0.13, *p* < 0.01). Mature coping style was positively correlated with resilience (*r* = 0.47, *p* < 0.01), while immature coping style was negatively correlated with resilience (*r*=-0.18, *p* < 0.01). Mature coping style was negatively correlated with immature coping style (*r*=-0.21, *p* < 0.01).

**Table 1:** Bi-variate correlations of the study variables

Variables	1	2	3	4	5
1. Unpredictability in childhood	—				
2. Mature coping style	-0.23^**^	—			
3. Immature coping style	0.13^**^	-0.21^**^	—		
4. Resilience	-0.18^**^	0.47^**^	-0.18^**^	—	
5. Depression	0.22^**^	-0.41^**^	0.50^**^	-0.32^**^	—

Note: Sample size *N* 601; ^*^p < 0.05 ,^**^p < 0.01,^***^p < 0.001

### Correlation between unpredictability in childhood and depression: test of serial mediating model

Following the mediation effect testing procedure [47, 48], this study delves into the mediating roles of mature coping style, immature coping style, and resilience in the relationship between unpredictability in childhood and depression. For a mediation effect to be deemed significant, it must satisfy three conditions: (1) The independent variable (e.g., unpredictability in childhood) must significantly impact the dependent variable (e.g., depression); (2) The independent variable (e.g., unpredictability in childhood) must significantly correlate to the mediating variables (e.g., mature coping style, immature coping style, and resilience); and (3) The mediating variables (e.g., mature coping style, immature coping style, and resilience) must in turn significantly impact the dependent variable (e.g., depression). Aside from gender, all variables were standardized. Data processing using Harman’s single-factor test revealed that common method variance did not pose a significant issue in this investigation.

Table 2 presents the results from the mediation of the mature coping style, immature coping style, and resilience in the relationship between unpredictability in childhood and depression. In the first step, unpredictability in childhood was found to have a significant and positive association with depression (*β* = 0.79, *t* = 5.43, *p =* 0.000). In the second step, mature coping style and unpredictability in childhood were observed to show a significant association with depression (*β*=-0.70, *t*=-8.71, *p =* 0.000); immature coping style and unpredictability in childhood were observed to show a significant association with depression (*β* = 0.61, *t* = 12.72, *p =* 0.000) In the third step, unpredictability in childhood, mature coping style, immature coping style, resilience and depression were observed to show a significant association with depression (*β*=-0.18, *t=*-3.25, *p =* 0.002).

**Table 2:** Depression model test

Variables	Step 1		Step 2		Step 3	
	β	t	β	t	β	t
Gender	-0.26	-0.36	-0.54	-0.91	-0.79	-1.34
Age	0.19	0.81	0.19	0.96	0.20	1.04
unpredictability in childhood	0.79	5.43^***^	0.33	2.71^**^	0.30	2.45^*^
Mature coping style			-0.70	-8.71^***^	-0.57	-6.49^***^
Immature coping style			0.61	12.72^***^	0.60	12.50^***^
Resilience					-0.18	-3.25^**^
*R²*	0.05		0.36		0.37	
*F*	10.24^***^		67.07^***^		58.56^***^	

Note: Sample size *N* 601; ^*^p < 0.05 ,^**^p < 0.01,^***^p < 0.001

Table 3 presents the results from the mediation of the mature coping style and immature coping style in the relationship between unpredictability in childhood and resilience. In the first step, unpredictability in childhood was found to have a significant and positive association with resilience (*β*=-0.46, *t*=-4.62, *p* = 0.000). In the second step, mature coping style and unpredictability in childhood were observed to show a significant association with resilience (*β* = 0.71, *t* = 11.79, *p =* 0.000); immature coping style and unpredictability in childhood were observed to show a significant association with resilience (*β*=-0.08, *t=*-2.08, *p* = 0.046).

**Table 3:** Resilience model test

Variables	Step 1		Step 2	
	β	t	β	t
Gender	-1.34	-2.71^**^	-1.44	-3.25^**^
Age	0.02	0.10	0.08	0.55
unpredictability in childhood	-0.46	-4.62^***^	-0.19	-2.02^*^
Mature coping style			0.71	11.79^***^
Immature coping style			-0.08	-2.08^*^
*R²*	0.04		0.25	
*F*	9.11^***^		38.53^***^	

Note: Sample size *N* 601; ^*^p < 0.05 ,^**^p < 0.01,^***^p < 0.001

Table 4 presents the results of the relationship between unpredictability in childhood and coping styles. For mature coping, unpredictability in childhood was found to have a significant and negative association with mature coping style (*β*=-0.36, *t=*-5.74, *p =* 0.000). For immature coping style, unpredictability in childhood was found to have a significant and positive association with immature coping style (*β* = 0.34, t = 3.26, *p =* 0.002). Therefore, mature coping style, immature coping style, and resilience played a serial mediating role between unpredictability in childhood and depression.

**Table 4:** Coping style model test

Variables	Mature coping		Immature coping	
	β	t	β	t
Gender	0.21	-0.68	0.70	1.36
Age	-0.10	-1.01	-0.11	-0.64
Unpredictability in childhood	-0.36	-5.74^***^	0.34	3.26^**^
*R²*	0.06		0.02	
*F*	11.79^***^		4.23^**^	

Note: Sample size *N* 601; ^*^p < 0.05 ,^**^p < 0.01,^***^p < 0.001

**Fig. 2:**
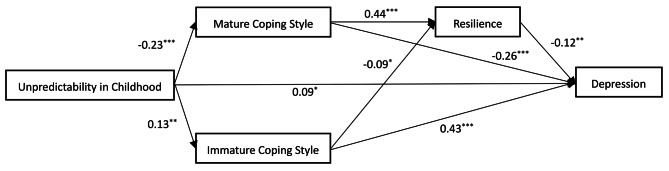
Model map of unpredictability in childhood and depression. Note: Sample size *N* 601; ^*^p < 0.05 ,^**^p < 0.01,^***^p < 0.001

As shown in Fig. 2, mature coping style played a mediating role between unpredictability in childhood and depression (effect = 0.21, effect ratio = 27%, 95% *CI* = 0.13–0.31). Immature coping style played a mediating role between unpredictability in childhood’s impact and depression (effect = 0.19, effect ratio = 24%, 95% *CI* = 0.08–0.33) Mature coping style and resilience serially mediate the relationship between unpredictability in childhood and depression (effect = 0.04, effect ratio = 5%, 95% *CI* = 0.02–0.08). Immature coping style and resilience serially mediate the relationship between unpredictability in childhood and depression (effect = 0.004, effect ratio = 1%, 95% *CI* = 0.001–0.02).

## Discussion

Although the link between unpredictability in childhood and depression has been extensively studied over the years, there has been little inquiry into how this effect was preceded, especially in the Chinese background. In this study, a serial mediation model showed how the relation between unpredictability in childhood and depression was funneled through mature coping style, immature coping style, and resilience.

Many studies have shown that unpredictability in childhood increases the risk of depression in adulthood [3, 9, 15], but few local studies in China have discussed this topic. The results of this study showed that under the Chinese cultural background, unpredictability in childhood was positively correlated with depression in college students, which was the same as the conclusion of previous studies in other countries, confirming that the relationship between unpredictability in childhood and depression is consistent across cultures. Some studies hold that the more times empirical studies repeat previous conclusions, the more reliable the research conclusions would be due to the generalization of the cumulative conclusions [49]. Therefore, the influence of unpredictability in childhood on depression should be highly emphasized.

Beck believed that depression could be explained by atavism in mechanism, which might be adaptive [10]. Nesse also believed that the survival function of depression might include strategies that push individuals to disengage themselves from unattainable goals and regulate their energy input patterns to achieve a survival advantage in certain environments [50]. According to the life history theory, since disasters and diseases often occur in unpredictable living environments, individuals are less likely to obtain returns from long-term investment, and individuals in such environments are more inclined to experience short-term satisfaction [22]. Therefore, depression was an adaptation to an unpredictable environment, which was consistent with the hypothesis of this study that individuals with unpredictable childhood (mainly including the unpredictable family environment and the unpredictable material environment) were more prone to depression. At present, few studies have explored the mechanism of unpredictability in childhood on depression. In this paper, three variables, mature coping style, immature coping style, and resilience, were introduced to further explore the mechanism of unpredictability in childhood on depression. This study shows that mature coping style, immature coping style, and resilience had a serial mediating effect between unpredictability in childhood and depression, which indicates that mature coping style, immature coping style, and resilience are important internal factors between unpredictability in childhood and depression.

Previous studies have shown that individuals whose early life was in an unpredictable environment tended to seek short-term gratification [22], which might lead these individuals to seek instant gratification [51]. When facing stressful events, they tended to use the immature coping style that could reduce the pain in a short time but not for a long time to solve the problem (e.g., fantasy and avoidance). At the same time, they seldom use mature coping styles (e.g., problem-solving and help-seeking) that can solve problems in the long run, which is consistent with the results of this study. Although few studies have explored whether unpredictability in childhood is correlated with coping styles, many studies have shown that adverse experiences and traumas in childhood affect individuals’ coping styles [52, 53]. Individuals living in families with high stress from childhood were more likely to adopt emotion-focused strategies and less likely to adopt problem-focused coping strategies when facing stress [52, 54]. In this study, unpredictability in childhood could be considered high stress and adverse experiences in childhood. In addition, mature coping styles and immature coping styles are similar to the emotion-oriented approach (e.g., self-accusation) and problem-solving-oriented approach (e.g., problem-solving). Therefore, this study claims that unpredictability in childhood could influence individuals’ mature coping style and immature coping style. At the same time, many past studies showed that an immature coping style was positively correlated with depression and that a mature coping style was negatively correlated with depression) [41, 55, 56], which was consistent with the results of this study. As a result, mature coping style and immature coping style mediate the relationship between unpredictability in childhood and depression.

Research has shown that resilience mediates the relationship between coping styles and depression. Previous studies found that coping styles in college students were developed from childhood, and coping styles in childhood would affect the application of different coping styles in the future life of individuals [57], which made the development of coping styles in childhood a key factor affecting the development of individuals’ physical and mental health [58]. Simultaneously, an immature coping style reduces the ability of individuals to cope with pressure and reduce their control over life, thus leading to low resilience. Individuals who adopt a mature coping style can solve problems through effective strategies, thus improving their resilience [59, 60]. Additionally, the increase in mature coping style and the decrease in immature coping style would also promote the development of a self-supporting personality, which would help people face life events with more flexibility. Eventually, this personality trait promotes the development of resilience [59]. With the improvement of resilience, individuals suffer fewer negative impacts when facing negative situations, thus reducing their risk of depression. Therefore, resilience mediates the relationship between immature coping style and depression. Resilience mediates the relationship between mature coping style and depression.

The serial mediation model proposed in this study provides an in-depth explanation of the mechanism of unpredictability in childhood on depression. This model shows that mature coping style, immature coping style, and resilience played serial mediating effects between unpredictability in childhood and depression. The results showed that the total mediating effect was 0.44, indicating that the mediating effect was of great significance in explaining the increased risk of depression caused by unpredictability in childhood. Second, the mediating effects of mature coping style and immature coping style were the largest, at 0.21 and 0.19, respectively. The serial mediating effect of unpredictability in childhood on depression through mature coping style and resilience was 0.04, and the serial mediating effect of unpredictability in childhood on depression through immature coping style and resilience was 0.004, indicating that the mediating effect was significant. It is worth noting that the serial mediating effect accounted for a relatively small proportion of the total effect, which does not mean that the serial mediating effect in this study is meaningless. First, small effect sizes can still have important theoretical implications. Many phenomena in social science are often affected by various factors. Social science theories usually only predict the influence of a certain factor and less predict the absolute size of the effect of a certain factor. In this case, small effects are also significant if they can support the theory [61]. Second, a small effect may also have important practical significance[63]. Methodologists believe that when a small effect might directly or indirectly lead to major results, a small effect might accumulate into a large effect over time. When a small effect affects a wide range of people, great attention should be given to a small effect even if it is small. In this study, although the serial mediating effect was relatively small, mature coping style, immature coping style, and resilience might lead to significant outcomes (such as depression). Additionally, the immature coping style and resilience caused by unpredictability in childhood might accumulate into a large effect. At the same time, immature coping styles and low resilience might exist in a large number of individuals, so this relatively small serial mediating effect should also be emphasized.

The findings from this study highlight the necessity for early, targeted interventions aimed at preventing depression among college students, particularly those with a history of childhood unpredictability. By implementing programs that enhance coping strategies and resilience, educators and mental health practitioners can significantly reduce the risk of depression. Such interventions could include workshops on effective problem-solving and emotional regulation techniques, tailored to the needs of students from diverse backgrounds. This approach not only addresses the immediate concerns related to depression but also contributes to long-term well-being.

### Limitations and future directions

The current study has several limitations. This investigation was a cross-sectional study and the participants were all from Jiangxi, which contributes to the persuasiveness of this study. Since some subjects with depression may experience distorted memories when recalling their childhood due to their symptoms, it is difficult to prove a causal relationship between variables. Future research can employ a longitudinal design to examine how developmental changes in depression are related to unpredictability in childhood. Secondly, this study verified the previous hypotheses, but the two variables of unpredictability in childhood and coping style can be subdivided and further explored. By subdividing these variables, we can better understand how different childhood environments affect depression in college students, thus providing different intervention methods for depression patients with different childhood experiences. Furthermore, the measures used in this study were self-reported and the results might be susceptible to response bias. Therefore, researchers could consider using multiple evaluations, from parents or teachers, in the future to verify our findings.

## Conclusion

The study was of great importance in exploring how unpredictability in childhood was related to depression in China, even if further replication and extension were needed. This study suggested that unpredictability in childhood was positively correlated with depression. Additionally, the present study indicates that mature coping style, immature coping style, and resilience play mediating roles in the relationship between unpredictability in childhood and depression independently and serially, which provides a new direction for exploring the cause of depression.

## Data Availability

The datasets used in this study are available from the corresponding author on reasonable request.
